# Supplementary material for: Modulation of Fat Deposition–Gut Interactions in Obese Mice by Administrating with Nobiletin
Source: Genes (Basel). 2023 May 10;14(5):1062. doi: 10.3390/genes14051062 (PMC10217980; doi:10.3390/genes14051062)
Supplement: Supplementary file 1 [file genes-14-01062-s001.zip › genes-2368843-supplementary.pdf]

Supplementary Information

Title: Modulation of fat deposition-gut interactions in obese mice by administrating with nobiletin

Cunzhen Zhao, Jiahua Guo, Chunyu Du, Yongjie Xu*

Supplementary Table1

Data preprocessing statistics and quality control

Sample Name	Raw PE	Combined	Clean Tags	Effective Tags	Base(nt)	AvgLen(nt)	GC%	Effective%
NC.1.1	84,132	83,237	81,586	64,621	27,113,546	420	51.77	76.81
NC.1.2	87,500	86,422	84,706	65,993	27,834,704	422	51.9	75.42
NC.1.3	99,814	99,011	97,063	68,778	28,735,062	418	54.16	68.91
NC.1.4	82,707	82,035	80,514	63,996	26,710,598	417	53.53	77.38
NC.1.5	87,021	86,270	84,709	61,195	25,760,411	421	53.49	70.32
NC.2.1	97,520	96,637	94,893	63,095	26,259,589	416	53.64	64.7
NC.2.2	97,445	95,434	93,387	62,356	26,164,022	420	52	63.99
NC.2.3	85,544	84,804	83,030	62,311	26,249,021	421	53.67	72.84
NC.2.4	87,175	86,418	85,028	62,694	25,986,157	414	54.38	71.92
NC.2.5	98,228	97,014	95,607	61,951	26,009,450	420	53.62	63.07
HF.1.1	82,051	80,931	79,246	60,688	25,488,673	420	51.02	73.96
HF.1.2	92,377	91,090	89,717	60,232	25,188,828	418	53.44	65.2
HF.1.3	85,036	84,104	82,772	63,193	26,457,082	419	53.08	74.31
HF.1.4	93,109	92,458	91,058	62,037	25,744,927	415	53.52	66.63
HF.1.5	83,844	82,812	81,542	69,218	28,632,262	414	51.93	82.56
HF.2.1	92,835	91,982	90,454	67,005	27,795,099	415	52.76	72.18
HF.2.2	59,344	58,961	58,038	42,934	17,774,144	414	53.71	72.35
HF.2.3	97,627	96,932	95,263	65,391	27,180,947	416	53.85	66.98
HF.2.4	90,620	89,950	88,593	65,965	27,140,962	411	54.22	72.79
HF.2.5	97,646	96,868	95,552	67,932	27,952,274	411	53.51	69.57
Average	89079	88169	86638	63079	26308888	417	53	71
